# Facial emotion processing and recognition among maltreated children: a systematic literature review

**DOI:** 10.3389/fpsyg.2014.01460

**Published:** 2014-12-17

**Authors:** Gabriela C. da Silva Ferreira, José A. S. Crippa, Flávia de Lima Osório

**Affiliations:** ^1^Department of Neurociences and Behavior, Medical School of Ribeirão Preto, University of São PauloRibeirão Preto, Brazil; ^2^Translational Medicine, National Institute of Science and TechnologyBrazil

**Keywords:** child, maltreatment, facial emotion, processing, recognition, review

## Abstract

Exposure to maltreatment is associated with biological, psychological, and social development impairments in children. This systematic literature review sought to determine whether an association exists between child maltreatment and facial emotion processing and recognition. The search was conducted using the databases PubMed, PsycINFO, and SciELO using the following keywords: “maltreatment,” “adversity,” “neglect,” “sexual abuse,” “emotional abuse,” “physical abuse,” “child^*^,” “early,” “infant,” “face,” “facial,” “recognition,” “expression,” “emotion^*^,” and “impairment.” Seventeen articles were selected and analyzed. Maltreated children tended to exhibit less accuracy in global facial tasks and showed greater reactivity, response bias, and electrophysiological activation of specific brain areas in response to faces expressing negative emotions, especially anger. We concluded that the results of this review are exploratory and non-conclusive due to the small number of studies published and the wide variety of aims and procedures. Those shortcomings notwithstanding, the results indicate definite tendencies and gaps that should be more thoroughly explored in future studies.

## Introduction

Child maltreatment encompasses any act of omission or commission by a parent or caregiver that results in harm or the potential for harm, regardless of intent (Gilbert et al., 2009). Currently, the types of child maltreatment most widely investigated are physical, emotional, and sexual abuse as well as neglect (Barnett et al., 1993).

Maltreatment during childhood is associated with several consequences that impair the biological, psychological, and social domains of human development (Kaufman et al., 2000; Cicchetti and Toth, 2005; McCrory et al., 2011b). With regard to the psychological and psychiatric domains, prospective and retrospective studies have found that child maltreatment is a risk factor for behavioral and mental disorders, including major depression and substance abuse, as well as personality, post-traumatic stress, and dissociative disorders (Cicchetti and Valentino, 2006; Gilbert et al., 2009; Scott et al., 2012).

Child maltreatment is also associated with deficiencies in social cognition, including the processing and recognition of facial emotion expressions (Cicchetti and Carlson, 1989; Gallese et al., 2004).

Psychobiological processes, which are modulated by different cerebral regions and neurocognitive systems, are understood as facial emotion processing when perceiving and assessing emotions. The processing of emotions, especially “basic” emotions (i.e., happiness, sadness, anger, fear, disgust, and surprise), involves several nervous system structures, especially the amygdala and prefrontal cortex. These neural substrates mature in parallel with development, yielding a greater refinement of emotional processing (Herba et al., 2006). In turn, the recognition of facial expressions involves the use of partial information based on the dynamic modulation of facial movements to generate a hypothesis concerning the emotion being expressed, which may be categorized and used to predict other people's behavior (Pollak and Sinha, 2002). Recognition, in addition to involving neurobiological processes, depends on neural experiences and learning, whereas the role of biological determinism and acquired experience in this skill have not been fully elucidated. Pollak and Kistler (2002). Rapid and precise emotional recognition represents a significant advancement in brain processing and child development that promotes better psychosocial adaptation (Gottman et al., 1975; Ekman, 1999; Pollak and Tolley-Schell, 2003).

Based on the assumption that children adjust their perceptual mechanisms to process the features that are most outstanding and familiar in their environments through the learning of social experiences, child maltreatment has been suggested to change sensory thresholds, causing less effective regulation, processing, and recognition of emotions (Pollak, 2008).

Because no thorough literature review has yet presented general conclusions on this subject and because the systematization of data in the literature is highly relevant, the present study provides a systematic literature review to establish the relationship between child maltreatment and the processing and recognition of facial expressions of emotion.

## Materials and methods

A systematic review of the literature was conducted according to the Cochrane protocol, without time limits, using the databases PubMed, PsycINFO, and SciELO with the following keywords: “maltreatment or adversity or neglect or sexual abuse or emotional abuse or physical abuse,” “child^*^ or early or infant,” “face or facial,” and “recognition or expression or emotion^*^ or impairment.” In addition, the references quoted by the selected articles were manually surveyed to broaden the scope of the review.

**Figure 1:**
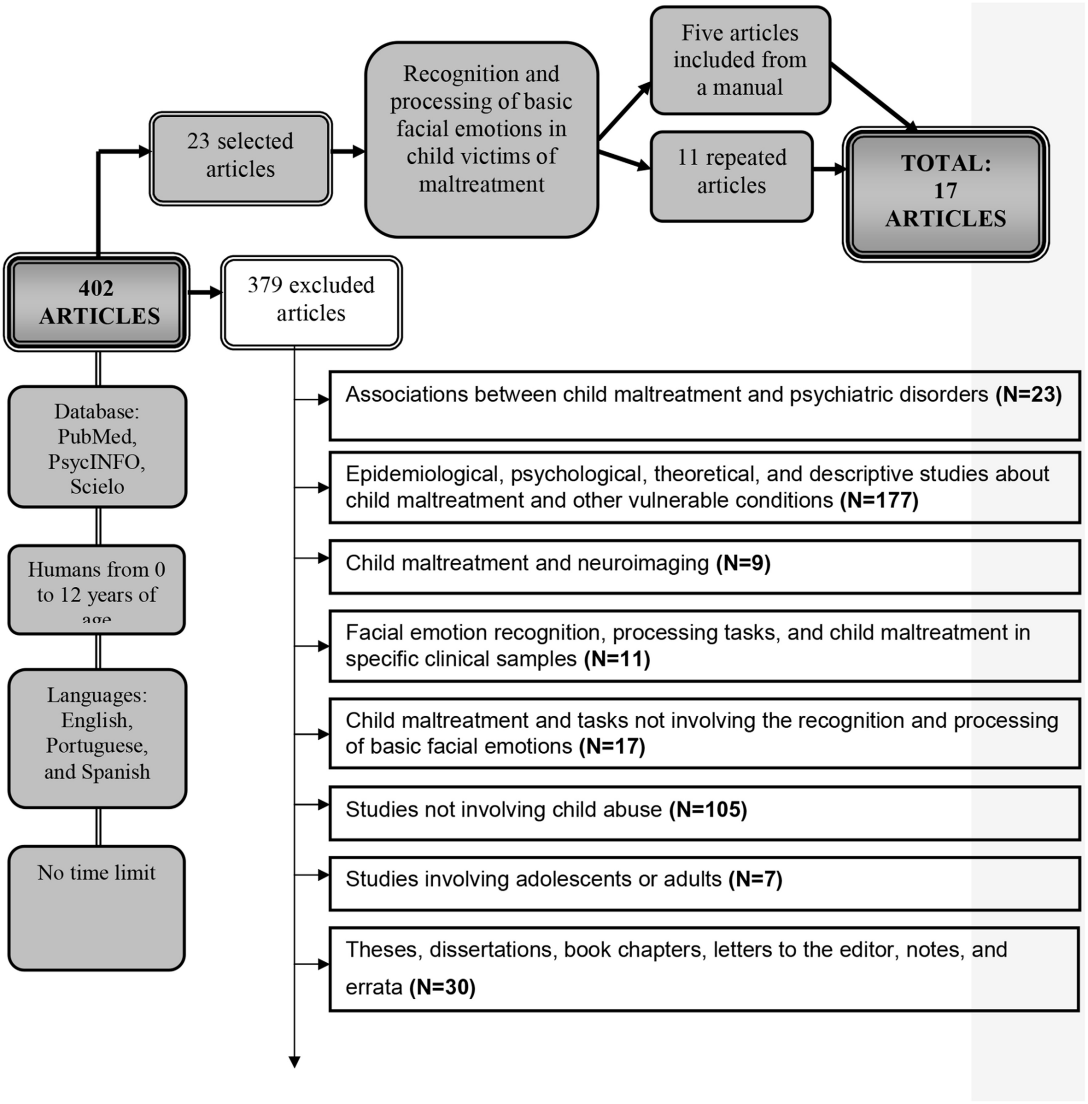
**Flowchart of article inclusion and exclusion**.

The process and criteria for article inclusion and exclusion are depicted in Figure 1.

## Results

Seventeen articles were included in the present review. Two psychologists with significant experience in this field assessed these articles for pertinence and compliance with the inclusion and exclusion criteria.

The search revealed that interest in the investigated topic began in the 1980s (*N* = 2), reached a peak in the 2000s (*N* = 9), and continues to the present (*N* = 3). The studies have been conducted in the United States (88%) and the United Kingdom (12%) only.

All studies exhibited the same methodological designs (i.e., case-control studies). The investigations could be classified in two groups based on their aims: the first group's primary aim was to assess the recognition of facial emotion, whereas the second group focused on facial emotion processing. Nine studies employed facial expression recognition tasks (Camras et al., 1983, 1988, 1990; During and McMahon, 1991; Pollak et al., 2000, 2009; Pollak and Kistler, 2002; Pollak and Sinha, 2002; Masten et al., 2008), whereas eight studies applied facial emotion processing tasks (Pollak et al., 1997, 2001; Pollak and Tolley-Schell, 2003; Cicchetti and Curtis, 2005; Pine et al., 2005; McCrory et al., 2011a, 2013; Curtis and Cicchetti, 2013).

### Sample characterization

The major sociodemographics analyzed are listed in Table 1.

**Table 1:** **Characterization of the samples**.

			**CLINICAL GROUP**				**CONTROL GROUP**		
**N° of article**	**Authors**	**Country**	**N (sex)**	**Age/years M (SD)**	**Type of maltreatment**	**Recruiting**	**N (sex)**	**Age/years M (SD)**	**Recruiting**
1^a^	Camras et al., 1983	US	17 (♂ = 11/♀ = 6)	5	NEG, UA	PPTPCA	17 (♂ = 11/♀ = 6)	5	School
2^a^	Camras et al., 1988	US	20 (♂ = 10/♀ = 10)	4.9	NEG, UA	PPTPCA	20 (♂ = 10/♀ = 10)	4.9	School
3^a^	Camras et al., 1990	US	20 (♂ = 10/♀ = 10)	4.9	NEG, PA	PPTPCA	20 (♂ = 10/♀ = 10)	4.9	School
4^a^	During and McMahon, 1991	US	23 (♂ = 15/♀ = 8)	4.9 (1.8)	NEG, PA	PPTPCA	23 (♂ = 13/♀ = 10)	5.2 (2.5)	Ad
5^b^	Pollak et al., 1997	US	23 (♂ = 18/♀ = 5)	9.2 (1.6)	NEG, PA	PPTPCA	21 (♂ = 17/♀ = 4)	9.2 (1.1)	GP
6^a^	Pollak et al., 2000	US	33 (♂ = 21/♀ = 12)	4.4	NEG, PA	PPTPCA	15 (♂ = 8/♀ = 7)	4.3 (0.5)	UPC
7^b^	Pollak et al., 2001	US	28 (♂ = 18/♀ = 10)	9.1 (1.7)	NEG, PA	PPTPCA	14 (♂ = 10/♀ = 4)	8.5 (1.6)	GP
8^a^	Pollak and Sinha, 2002	US	24 (♂ = 17/♀ = 7)	9.3 (1.6)	PA	PPTPCA	23 (♂ = 16/♀ = 7)	9.4 (1.5)	PPTPCA
9^a^	Pollak and Kistler, 2002	US	23 (♂ = ?/♀ = ?)	9.25	PA	PF/SWA	17 (♂ = ?/♀ = ?)	9.25	PPTPCA
10^b^	Pollak and Tolley-Schell, 2003	US	14 (♂ = 8/♀ = 6)	10.1 (1.2)	PA	DHS	14 (♂ = 9/♀ = 5)	10 (1.1)	Ad
11^b^	Cicchetti and Curtis, 2005	US	35 (♂ = 16/♀ = 19)	2.6 (0.15)	NEG, PA, SA	DHS	24 (♂ = 15/♀ = 9)	2.5 (0.1)	CSS
12^b^	Pine et al., 2005	US	34 (♂ = 15/♀ = 19)	10.3 (1.8)	DV	DCFS	21 (♂ = 7/♀ = 14)	9.9 (1.8)	DCFS
13^a^	Masten et al., 2008	US	29 (♂ = 14/♀ = 15)	11.3 (1.4)	NEG, PA, SA	PPTPCA	17 (♂ = 7/♀ = 10)	12 (2.0)	GP
14^a^	Pollak et al., 2009	US	49 (♂ = 25/♀ = 24)	9.5 (0.1)	PA	PPTPCA	46 (♂ = 23/♀ = 23)	9.5 (0.1)	GP
15^b^	McCrory et al., 2011a	UK	20 (♂ = 14/♀ = 6)	9.5 (1.4)	NEG, PA, SA, EA	CSS	23 (♂ = 11/♀ = 12)	12.5 (1.17)	School/Ad
16^b^	McCrory et al., 2013	UK	18 (♂ = 12/♀ = 6)	12.1 (1.4)	PA, DV	CSS	23 (♂ = 11/♀ = 12)	12.5 (1.2)	School/Ad
17^b^	Curtis and Cicchetti, 2013	US	25 (♂ = 12/♀ = 13)	1.3 (0.05)	NEG, PA, EA	DHS	20 (♂ = 8/♀ = 12)	1.3 (0.1)	CSS

a Focus of the study: facial emotion processing.

b Focus of the study: facial emotion recognition;

US, United States; UK, United Kingdom; N, number of participants in the sample; M, mean; SD, standard deviation; ♂, male; ♀, female; NEG, neglect, PA, physical abuse, SA, sexual abuse; EA, emotional abuse; DV, domestic violence; UA, unspecified abuse; PPTPCA, protection, prevention, and treatment programs for child abuse; PF/SWA, psychiatric facility/ social welfare agency; DHS, Department of Human Services; DCFS, Department of Children and Family Services; CSS, Community social services; GP, general population; UPC, university pediatric clinic.

The clinical and control groups were homogeneous with regard to gender, age, and social condition across all studies. On average, 25.5 children (median = 23) participated in the clinical samples, and 21 children (median = 20) participated in the controls. All studies included males and females. The average ages of the clinical groups and controls were 7.3 and 7.6 years, respectively.

Most clinical group volunteers were recruited from prevention, treatment, or protection against child maltreatment programs, departments of human services, or community-based social services. The control group volunteers were recruited from the general population from schools, via advertising in newspapers, Internet resources, and community-based services, among others.

The major inclusion criterion in all of the studies was the presence or absence of global or specific child maltreatment. The assessment and confirmation of child maltreatment were based on an analysis of clinical and legal reports at institutions or services specializing in maltreatment by professionals, investigators, or specialists. In the analysis, or to complement the available data, the authors used several instruments including guidelines and scales (see Table 2).

**Table 2:** **Sources of the research and the evaluation instruments used to detect child abuse**.

**Source/major instruments**	**Clinical and legal reports of maltreatment from a specialized institution or service (N = 17)^1,2,3,4,5,6,7,8,9,10,11,12,13,14,15,16,17^**
Source/complementary instruments (^*^)	The Guidelines of Manly, Cicchetti and Barnett, 1991 (*N* = 5)^5,6,7,11,17^
	Parent–Child Conflict Tactics Scale (*N* = 3)^8,10,14^
	Dunedin Abuse Scales (*N* = 2)^15,16^
	Child Bad Experience Quesntionnaire (*N* = 2)^15,16^
	The Guidelines of Kaufman et al., 1994 (*N* = 2)^15,16^
	The Guidelines of Guyer et al., 2006 and Kaufman et al., 2000 (*N* = 1)^13^
	Child Abuse Potential Inventory (*N* = 1)^4^
	Child Trauma Questionnaire in an adolescente psychiatric population (*N* = 1)^12^
	Standardized Questionnaire Assessing Level of Domestic Violence (*N* = 1)^12^
	State-Trait Anger Expression Inventory (*N* = 1)^14^
	Self-report of children (*N* = 1)^12^
	Interviews with parents (*N* = 1)^5^
	Maternal Maltreatment Interview (*N* = 1)^17^

* *Nonexclusive category; N, number of studies; superscript references based on Table 1*.

Approximately 47% of the studies (*N* = 8) did not report any exclusion criteria (Camras et al., 1983, 1988, 1990; Pollak and Sinha, 2002; Pollak and Tolley-Schell, 2003; Pine et al., 2005; Masten et al., 2008; Pollak et al., 2009). Of those that did, the most frequently stated were the presence or absence of specific abuse types, the presence of intellectual or learning disorders, residences at institutions or in unstable households, pregnancy complications, diagnoses of acquired immunodeficiency syndrome (AIDS), and children or mothers with psychotic disorders.

### Procedure characterization

As mentioned above, the studies included in the present review pursued two separate aims and therefore used different techniques. Thus, the procedures described in the present review take this division into consideration.

The major features of the various procedures are described in Table 3.

**Table 3:** **Major features of the procedures used in the facial emotion tasks**.

	**Recognition task (**N** = 9)**	**Processing task (**N** = 8)**
Task	Identify emotion (*N* = 9)^1,2,3,4,6,8,9,13,14^	Identify target (*N* = 5)^5,7,10,12,16^
		View faces (*N* = 2)^11,17^
		Identify gender (*N* = 1)^15^
Type of stimuli	Verbal history+facial photographs (*N* = 4)^1,2,3,6^	Paired images of emotions+target (*N* = 5)^5,7,10,12,16^
	Dynamic facial images on screen (*N* = 4)^8,9,13,14^	Pictures of facial emotions (*N* = 3)^11,15,17^
	Name of emotion+facial photographs (*N* = 1)^4^	
Set of images/stimuli	Standardized: yes (*N* = 9)^1,2,3,4,6,8,9,13,14^	Standardized: yes (*N* = 8)^5,7,10,11,12,15,16,17^
	N° of stimuli: mean = 77.25	N° of stimuli: mean = 304.3
	median = 31	median = 160
	Color: black/white (*N* = 7)^1,2,3,4,6,9,13^	Color: black/white (*N* = 8)^5,7,10,11,12,15,16,17^
	color (*N* = 2)^8,14^	
	N° of participants: mean = 8.25	N° of participants: mean = 25.5
	median = 8	median = 3
	Participant age: child (*N* = 4)^1,2,3,4^	Participant age: adult (*N* = 8)^5,7,10,11,12,15,16,17^
	adult (*N* = 4)^8,9,13,14^	
	not specified (*N* = 1)^6^	
	Participant gender: female (*N* = 1)^4^	Participant gender: female (*N* = 4)^5,7,11,17^
	female/ male (*N* = 8)^1,2,3,6,8,9,13,14^	female/male (*N* = 3)^10,12,16^
		not specified (*N* = 1)^15^
	Evaluated emotions(^*^): happiness (*N* = 9)^1,2,3,4,6,8,9,13,14^	Evaluated emotions(^*^): anger (*N* = 8)^5,7,10,11,12,15,16,17^
	fear (*N* = 9)^1,2,3,4,6,8,9,13,14^	happiness (*N* = 7)^5,7,10,11,12,16,17^
	sadness (*N* = 8)^1,2,3,4,6,8,9,14^	neutral (*N* = 7)^5,10,11,12,15,16,17^
	anger (*N* = 8)^1,2,3,4,6,8,9,14^	fear (*N* = 2)^7,15^
	disgust (*N* = 5)^1,2,3,4,6^	
	surprised (*N* = 5)^1,2,3,4,14^	
	neutral (*N* = 1)^13^	
Outcomes(^*^)	Accuracy (*N* = 7)^1-4,6,8,13^	Latency time of target/gender (*N* = 6)^5,7,10,12,15,16^
	Intensity/ distinctness (*N* = 4)^8-9,13,14^	EEG: amplitude and/or latency (*N* = 5)^5,7,10,11,17^
	Response bias (*N* = 3)^6,8,14^	Accuracy of target/gender (*N* = 5)^5,7,10,12,15^
		fMRI: amygdala and anterior insula activation (*N* = 2)^15,16^
		Attention bias (*N* = 2)^12,16^

* Nonexclusive category; N, number of studies; superscript references based on Table 1; EEG, electroencephalogram; fMRI, Functional magnetic resonance imaging.

As Table 3 shows, the wide diversity and remarkable specificity of the procedures used are worthy of attention because no standard procedure was applied to investigate facial expression recognition or processing.

Although the tasks in all nine studies investigating facial emotion recognition consisted of identifying emotions from pictures, they used different photographs or dynamic images; others presented verbal stories or emotion-evoking words. The eight studies that investigated facial emotion processing sought to focus the volunteers' attentions on faces, which were most often displayed quickly and in pairs. For this purpose, most studies (Pollak et al., 1997, 2001; Pollak and Tolley-Schell, 2003; Pine et al., 2005; McCrory et al., 2013) requested the volunteers press a button whenever they identified a target appearing on the faces, whereas the other three studies requested that volunteers identify the face's gender or passively look at the stimuli.

The stimuli used in all studies were standardized (Ekman and Friesen, 1975, 1976, 1978), and most consisted of black-and-white pictures of male and female adults. Nevertheless, the numbers of image subjects, stimuli, and the types of tasks exhibited remarkable variation among the studies.

Wide variety was also found with regard to the assessed emotions; happiness and anger were most frequently investigated in both types of studies.

### Outcomes

The major results of the studies are described in Table 4, considering their aims and outcomes.

**Table 4:** **Major results of the studies with regard to the outcomes**.

	**ACCURACY**		**INTENSITY/ LATENCY TIME**		**BIAS**	**EEG**	**fMRI**
	**Total**	**Specific faces**	**Total**	**Specific faces**			
Recognition Task	MT = C	Anger	**Intensity**	**Intensity**	**Response Bias**		
(*N* = 9)^*^	(*N* = 2)^8,13^	MT = C	MT < C	Anger	Anger		
	MT < C	(*N* = 2)^1,8^	(*N* = 1)^13^	MT < C (*N* = 3)^8,9,14^	MT = C (*N* = 2)^8,14^		
	(*N* = 5)^1,2,3,4,6^	MT < C (*N* = 1)^6^		Fear	MT > C (*N* = 1)^6^		
		Disgust		MT = C (*N* = 3)^8,9,14^	Sadness		
		MT = C (*N* = 1)^1^		MT < C (*N* = 1)^13^	MT = C (*N* = 2)^8,14^		
		MT < C (*N* = 1)^6^		Sadness	MT > C (*N* = 1)^6^		
		Sadness		MT = C (*N* = 2)^9,14^			
		MT = C		MT > C (*N* = 1)^8^			
		(*N* = 2)^1,8^					
		MT < C (*N* = 1)^6^					
Processing Task	MT = C	Anger	**Latency time**	**Latency time**	**Attention Bias**	**Latency**	**Right amygdala:**
(*N* = 8)^*^	(*N* = 3)^5,7,10^	MT < C	MT = C	Anger:	Anger	MT = C(*N* = 4)^5,7,10,11^	Anger Neutral: MT > C
	MT < C	(*N* = 2)^7,15^	(*N* = 4)^7,10,15,16^	MT = C	MT = C (*N* = 1)^16^	**Amplitude**	(*N* = 2)^15,16^
	(*N* = 2)^12,15^	Fear:	MT > C (*N* = 1)^5^	(*N* = 6)^5,7,10,12,15,16^	MT (physical): ↑MT severity	Anger	Happiness/Neutral: MT >
		MT = C		Fear	severity↑stimulus	MT = C (*N* = 4)^5,10,11,17^	MT = C (*N* = 1)^16^
		(*N* = 2)^7,15^		MT = C (*N* = 2)^7,15^	avoidance (*N* = 1)^12^	MT > C (*N* = 3)^7,10,11^	**Left amygdala:**
		Happiness		Happiness	MT (neglect): no	MT < C (*N* = 1)^17^	Anger /Sadness: MT > C
		MT = C (*N* = 1)^7^		MT = C (*N* = 5)^5,7,10,12,16^	correlation (*N* = 1)^12^	Happiness	MT = C (*N* = 1)^15^
		Neutral		Neutral	Happiness	MT < C (*N* = 5)^5,7,10,11,17^	**Amygdala:**
		MT = C (*N* = 1)^15^		MT = C	MT = C (*N* = 1)^16^	MT > C (*N* = 1)^17^	Anger: ↑activation ↑MT
				(*N* = 5)^5,10,12,15,16^	MT (physical,	MT < C (*N* = 1)^17^	precocity (*N* = 1)^16^
					neglect):		**Left anterior insula:**
					no correlation		Anger/Sadness: MT > C
					(*N* = 1)^12^		(*N* = 1)^15^
					Neutral		Anger: ↑activation ↑MT
					MT = C (*N* = 1)^16^		severity (*N* = 1)^15^
							**Bilateral anterior insula:**
							Anger /Neutral: MT > C
							(*N* = 1)^15^

^*^Nonexclusive category; N, number of studies; superscript references based on Table 1; MT, maltreatment group; C, control group; EEG, electroencephalogram; fMRI, Functional magnetic resonance imaging.

With regard to accuracy in the recognition task, 71% of the studies (Camras et al., 1983, 1988, 1990; During and McMahon, 1991; Pollak et al., 2000) found that global facial expression recognition was impaired in children who were maltreated, whereas only one (Pollak et al., 2000) of three studies found impairments in the recognition of specific (especially negative) emotions. Thus, these results are contradictory.

With regard to the assessment of global facial expression processing, 60% of studies did not find evidence of impairment (Pollak et al., 1997, 2001; Pollak and Tolley-Schell, 2003). The two studies (Pollak et al., 2001; McCrory et al., 2011a) that assessed specific emotion processing also found no differences in the results. Although the studies employed a wide variety of procedures, a global qualitative analysis indicated that they did not directly influence the results of accuracy evidenced in studies, because no specific resultant trend was observed when the findings were assessed with the parameter being the different procedures used. However, regarding the sociodemographic characteristics of the samples, the following resultant trend was observed: the studies conducted with younger children (average age range = 4.4–10.3 years old) found that the performance of maltreated children was poorer than that of the controls. This difference was not found in studies conducted with older subjects (average age range = 9.1–11.3 years).

A second set of outcomes included the intensity or sharpness of faces in the recognition task and the latency times of the processing task. The intensity necessary for the global identification of emotions, evaluated by the morphing techniques, was not significantly different between groups. However, three out of the four studies (Pollak and Kistler, 2002; Pollak and Sinha, 2002; Pollak et al., 2009) investigating the recognition of specific facial expressions found that the emotional intensity required by maltreated children to recognize faces expressing anger was lower compared with the controls; thus, maltreated children are more reactive.

No study found differences in response latency or target identification between maltreated children and controls with regard to the processing of either global or specific tasks.

Three out of the five studies (Pollak and Sinha, 2002; Pollak et al., 2009; McCrory et al., 2013) that assessed response and attention bias did not find differences between maltreated children and controls, whereas the other two studies found interesting differences. Pollak et al. (2000) showed that maltreated children tended to identify emotions such as sadness and anger more frequently. Pine et al. (2005) investigated attention bias and found that higher levels of abuse predicted the tendency to avoid angry faces during tasks.

Five facial expression-processing studies used electroencephalography (EEG) to assess event-related potentials (ERPs): electrical potentials in the brain directly related to the presentation of a stimulus. In particular, 60% of those studies (Pollak et al., 2001; Pollak and Tolley-Schell, 2003; Cicchetti and Curtis, 2005) found greater wave amplitudes among maltreated children when processing angry faces.

Two studies (McCrory et al., 2011a, 2013) used functional magnetic resonance imaging (fMRI) to assess the activity of specific brain areas and found that the amygdala and insula were the most active regions during the processing of anger. These studies also showed that the level of amygdala activation during the processing of angry faces was negatively associated with onset age of abuse (McCrory et al., 2013). Furthermore, the activation level of the left anterior insula was associated with abuse severity (McCrory et al., 2011a).

## Discussion

### Critical findings

The results revealed that few studies have been conducted thus far in this specific field, and the number of consistent findings in the present review is relatively small. In addition, most such studies have been conducted in developed countries with better psychosocial and economic conditions compared with the rest of the world, which might have influenced the occurrence and experience of abuse. Therefore, because the findings of the present review apply to specific social conditions, they cannot be generalized to developing countries. Thus, studies with children from different countries and subjected to different social conditions are necessary.

In this regard, Garbarino and Kostelny (1992) conducted a study in the United States that indicated the strong influence exerted by socioeconomic and demographic factors on the rates of child maltreatment. Furthermore, this study demonstrated that areas at high risk for child abuse are characterized by poor social cohesion and disorganization as well as by a lack of resources and social structure. Similarly, Euser et al. (2011) investigated immigrant families in the Netherlands and found that low parental educational levels were a risk factor for child maltreatment.

The samples in the current review were relatively small, and the specificity of the various types of child maltreatment was given little attention: 41.2% of the studies (Camras et al., 1983, 1988; Pollak and Sinha, 2002; Pollak and Kistler, 2002; Pollak and Tolley-Schell, 2003; Pine et al., 2005; Pollak et al., 2009) approached child abuse as a single construct or did not separately assess each type. This fact denotes a significant methodological limitation because (although the various types of maltreatment are often concomitant and interrelated) according to the literature, each type of abuse induces different effects on child development and adjustment in adult life (Higgins and McCabe, 2000; Lee and Hoaken, 2007). Because emotion processing and recognition might bear specific patterns of correlation with various types of abuse, future studies should incorporate this possibility in their methodological designs.

The results revealed that previous studies have employed a wide variety of procedures; a single methodological standard to investigate the recognition and processing of facial expressions is not yet available. Thus, the large diversity of adopted procedures hampers more precise comparisons and conclusive results, such as is the case of those from meta-analytical studies. Although qualitative data analysis does not point toward a bias of results as a function of the used procedures, the different methodological variables are known to influence this process.

Specifically, the literature indicates that task performance is often influenced by variables related to the respondents and the procedures applied. Among these influential factors, the following stand out: (a) type of task; (b) task demand level; and (c) respondent age and gender (Durand et al., 2007).

The study conducted by Bruce et al. (2000) likely illustrates the relevance of the task demand level. In this study, when children were asked to perform a less complex task, in which they had to choose which of two presented faces expressed the emotion verbally expressed by the evaluator, high accuracy was obtained from 6 years of age. However, in a more complex task, in which children had to select which of two faces represented the same emotion as a third face (i.e., without a verbal stimulus), a good level of accuracy was achieved only from 10 years of age.

With regard to respondent characteristics, the literature review conducted by McClure (2000) showed that girls perform better than boys in emotion processing and recognition from childhood through adolescence, which is due to a sum of factors, including cultural developmental and neurological maturation aspects (McClure, 2000). Similarly, older children exhibit better emotion processing and recognition skills (Herba et al., 2006). Two of the studies included in the present review (During and McMahon, 1991; Pollak et al., 1997) compared children distributed across two age ranges and found that accuracy was greatest among those who were older and maltreated.

Regarding the influence of those variables on the results of the present review, approximately half of the studies (Camras et al., 1983; During and McMahon, 1991; Pollak et al., 1997, 2000, 2001; Pollak and Sinha, 2002; Pollak and Kistler, 2002; McCrory et al., 2011a, 2013) analyzed did not consider the demographic aspects in a more discerning manner, as the samples were not paired according to participant gender and/or this variable was not analyzed separately, which is one of the limitations of the studies. On the other hand, the variable age was associated with different results, as studies whose samples included younger children showed that abused children tend to present higher error rates on tasks compared with the control group. However, no difference between the hit/error rates of abused and non-abuse children was found in studies whose samples included older children. In this sense, it is possible that children who are victimized at early ages present greater impairments in social cognition, which may be compensated for or even healed over the course of the development with the maturation of biological structures (Grady, 2002; McClure, 2000). However, for this statement to sustain itself more precisely, it would be necessary to know the moments in life when the trauma was experienced, as well as the time interval between the trauma and the assessment, so the influence of those variables on the findings could be discarded. Unfortunately, this information was not presented by the great majority of the analyzed studies, impeding this analysis.

One of the major findings of this review was regarding the accuracy rate, where most of the studies (*N* = 7) showed that abused children have a greater hit rate when different emotions are considered together. This finding may be associated with neurobiological, environmental, and learning aspects.

With regard to neurobiological factors, the literature indicates that major stressors during child development can harm cognitive functions and the development of significant brain areas (Teicher, 2000; Teicher et al., 2003; Lee and Hoaken, 2007). Because chronic stressors activate brain areas such as the limbic system and prefrontal cortex, their connections might be strengthened at the expense of other significant neural connections, such as those responsible for regulating emotion (which then become underused; Lee and Hoaken, 2007). The neurobiological changes exhibited by maltreated children are likely associated with the brain areas involved in processing and recognizing the facial expression of basic emotions, such as the amygdala and prefrontal cortex (Herba et al., 2006).

From the environmental and learning point of view, it is hypothesized that different family environments experienced by abused children may influence the ease of recognition of specific emotions. Depending on the family environment, some types of emotions would be more experienced and expressed and, hence, more easily recognized, while other types of emotions (less familiar) would have their recognition hampered. This hypothesis supports the results from the study by Pollak et al. (2000), which is included in this review.

Notably, this was the only study to show that abused children present more errors while recognizing faces with negative emotions and the only to evidence specificities on the answers as a function of the type of trauma experienced by the children. Children who are victims of negligence present more difficulties in detecting negative emotions, such as anger, compared with the control group, whereas children who are victims of physical abuse present more difficulties detecting negative emotions, such as sadness and disgust. These results have not been presented by studies other than the present review, and they indicate the following relevant aspect: different types of traumas may be associated in a specific manner to different impairments in recognizing facial emotions. Thus, it is important that researchers consider the impact of each variable alone.

With regard to the relevance of the environment, MacMillan et al. (2009) performed a literature review of child maltreatment prevention programs and found that certain types of social, therapeutic, and family interventions (e.g., home visits, protection programs, and child institutionalization) may be beneficial by minimizing the recurrence of abuse and its damaging consequences as well as by improving the adjustment in adult life (MacMillan et al., 2009).

The present review also found differences between maltreated children and controls regarding the intensity of emotion necessary for anger recognition. These results corroborate those of Pollak (2008) who found that the major perceptual alterations exhibited by maltreated children are more related to anger than other emotions.

In the present review, an assessment of the intensity required to recognize facial emotions in dynamic tasks showed that children (abused) best recognize faces expressing anger with the lowest level of available information, compared with the control group. These results suggest that children subjected to maltreatment might be hypervigilant and more reactive to signs of anger. In turn, this might affect their development by increasing their level of anxiety and their predisposition to aggressive reactions (Shackman et al., 2007; Pollak, 2008). Hypervigilance might be due to the exposure of these children to high levels of anger and hostility in their family environments; thus, they are able to detect these emotions more rapidly than controls. Although hypervigilance is a maladaptive behavior in normal environments, the early (and accurate) recognition of threats is a valuable ability in hostile environments because it allows children to anticipate signs of conflict and makes them more sensitive to signs of anger.

Nevertheless, the emotion of anger was not systematically accurate. One of the studies that assessed response and attention bias found that the maltreated group exhibited a greater tendency to attribute anger and sadness to faces with other basic emotions, and another study reported that higher levels of abuse predicted the tendency to avoid anger stimuli during the task. Similarly, one study conducted on a sample of adolescents found that exposure to severe abuse was associated with the better detection of and greater response bias regarding anger (Gibb et al., 2009).

Previous authors have speculated that response biases might be caused by learning and the emotional experiences to which children are exposed in the family environment. This effect might occur when the parents' or family's ability to express emotions is limited or highly aggressive, thereby impairing the interpretation of emotions in general (Dunn et al., 1991a,b).

According to the literature, the neurological processing of anger and fear is closely related to a system that involves the amygdala and related areas such as the thalamus, insula, rostral anterior cingulate, and prefrontal cortex (Keightley et al., 2003).

The neurophysiological data reported by the studies included in the present review indicate that the perception of angry faces is associated with increased EEG wave amplitude and greater fMRI activation of brain areas such as the right and left amygdala and the bilateral and left anterior insula.

The amygdala likely plays relevant roles in fear conditioning, aggression control, emotional memory, and the fight-or-flight response, whereas excessive activation in response to negative stimuli is associated with anxious traits, post-traumatic stress disorder, major depression, and cognitive bias (Edwards et al., 2003; Dannlowski et al., 2012). As evidenced by the two studies in this review (McCrory et al., 2011a, 2013) that performed neuroimaging tests, greater alterations were found in the amygdala, thereby supporting findings that show that the amygdala is one of the brain structures that undergoes changes due to early stress and child maltreatment (McClure, 2000; Teicher et al., 2003).

One of the studies (McCrory et al., 2011a) included in the present review also found that the insula changes in response to anger. Two fMRI studies using facial expression processing tasks possibly elucidated this finding because they identified increased activation in the anterior insula and amygdala in response to anger stimuli among individuals with anxiety disorders and soldiers deployed to combat zones (Etkin and Wager, 2007; van Wingen et al., 2011). Based on the greater activation that occurs under conditions favorable to hypervigilance, one might assume that these brain areas help to predict and adapt to threats. Similarly, electrophysiological studies have indicated the presence of selective hypervigilance to anger stimuli among children subjected to physical abuse, which is associated with high levels of anxiety (Pollak, 2008).

The fMRI studies also found that the activation of the amygdala and anterior insula in response to angry faces was correlated with earlier onset and greater severity of maltreatment. In addition, other studies found that onset age and maltreatment length might increase developmental impairments (Lee and Hoaken, 2007; Shackman et al., 2007). Thus, future studies should address this issue more thoroughly by including the onset age and maltreatment length, which, as highlighted previously, were neither evaluated nor carefully measured in the studies of the present review.

### Implications for practice, policy, and research

This first systematic literature review on the current subject was exploratory and inconclusive due to the aforementioned limitations, the methodological diversity among studies, and sample restrictions in particular. These limitations notwithstanding, the studies showed that maltreated children are less accurate than controls with regard to global facial recognition and processing tasks. In addition, these children exhibited greater reactivity, response biases, and electrophysiological activation of the amygdala and anterior insula to faces expressing negative emotions, especially anger.

Additional studies are needed that (a) apply standard procedures to control the variables that influence facial expression recognition and processing; (b) include large samples that represent different social contexts; (c) analyze the effect of the specific types of maltreatment; (d) measure and control relevant variables such as onset age and maltreatment length; and (e) map the effect of protective factors such as participation in preventive and therapeutic programs.

### Conflict of interest statement

The authors declare that the research was conducted in the absence of any commercial or financial relationships that could be construed as a potential conflict of interest.
